# Role of 14-3-3 protein family in the pathobiology of EBV in immortalized B cells and Alzheimer’s disease

**DOI:** 10.3389/fmolb.2024.1353828

**Published:** 2024-07-31

**Authors:** Prankur Awasthi, Dhruv Kumar, Saba Hasan

**Affiliations:** ^1^ Amity Institute of Biotechnology, Amity University Uttar Pradesh, Lucknow, India; ^2^ School of Health Sciences and Technology, UPES University Dehradun, Dehradun, India

**Keywords:** EBV, Alzheimer’s disease, DEGs, miRNA, 14-3-3 protein

## Abstract

**Background and Aims:**

Several studies have revealed that Epstein-Barr virus (EBV) infection raised the likelihood of developing Alzheimer’s disease (AD) via infecting B lymphocytes. The purpose of the current investigation was to assess the possible association between EBV infection and AD.

**Methods:**

The microarray datasets GSE49628, GSE126379, GSE122063, and GSE132903 were utilized to extract DEGs by using the GEO2R tool of the GEO platform. The STRING tool was used to determine the interaction between the DEGs, and Cytoscape was used to visualize the results. The DEGs that were found underwent function analysis, including pathway and GO, using the DAVID 2021 and ClueGo/CluePedia. By using MNC, MCC, Degree, and Radiality of cytoHubba, we identified seven common key genes. Gene co-expression analysis was performed through the GeneMANIA web tool. Furthermore, expression analysis of key genes was performed through GTEx software, which have been identified in various human brain regions. The miRNA–gene interaction was performed through the miRNet v 2.0 tool. DsigDB on the Enrichr platform was utilized to extract therapeutic drugs connected to key genes.

**Results:**

In GEO2R analysis of datasets with |log2FC|≥ 0.5 and *p*-value <0.05, 8386, 10,434, 7408, and 759 genes were identified. A total of 141 common DEGs were identified by combining the extracted genes of different datasets. A total of 141 nodes and 207 edges were found during the PPI analysis. The DEG GO analysis with substantial alterations disclosed that they are associated to molecular functions and biological processes, such as positive regulation of neuron death, autophagy regulation of mitochondrion, response of cell to insulin stimulus, calcium signaling regulation, organelle transport along microtubules, protein kinase activity, and phosphoserine binding. Kyoto Encyclopedia of Genes and Genomes analysis discovered the correlation between the DEGs in pathways of neurodegeneration: multiple disease, cell cycle, and cGMP-PKG signaling pathway. Finally, YWHAH, YWHAG, YWHAB, YWHAZ, MAP2K1, PPP2CA, and TUBB genes were identified that are strongly linked to EBV and AD. Three miRNAs, i.e., hsa-mir-15a-5p, hsa-let-7a-5p, and hsa-mir-7-5p, were identified to regulate most of hub genes that are associated with EBV and AD. Further top 10 significant therapeutic drugs were predicted.

**Conclusion:**

We have discovered new biomarkers and therapeutic targets for AD, as well as the possible biological mechanisms whereby infection with EBV may be involved in AD susceptibility for the first time.

## 1 Introduction

Alzheimer’s disease (AD) that usually affects old persons and is characterized by deterioration in the function of cognition along with a depreciation in the function of the motor nerve. The primary pathogenic characteristics are the accumulation of hyperphosphorylated tau generating neurofibrillary tangles within the cell with neuroinflammation, gliosis, and neurodegeneration, and the building up of amyloid-beta peptides producing plaques outside the cell (Talwar et al., 2018; Allnutt et al., 2020; Andrews et al., 2020). It is possible that viral infections pose a hazard for developing AD (Kittur et al., 1992). According to research, different viruses may use distinct routes and possess varying distributions throughout the brain. Additionally, substances connected to infections can infiltrate the old person’s brain, causing chronic inflammation that eventually results in neuronal degeneration (Carbone et al., 2014; Talwar et al., 2018). Kristin Levine and colleagues discovered that viral infections were linked to a higher risk of many neurodegenerative illnesses in a recent study that involved data mining from medical records (Levine et al., 2023). They found association between six neurodegenerative diseases—AD, amyotrophic lateral sclerosis (ALS), multiple sclerosis (MS), generalized dementia, Parkinson’s disease (PD), and vascular dementia—and multiple viral pathogens. The correlation among AD and viral encephalitis was the greatest. They also confirmed that 22 of the 45 viral exposures were found to be substantially linked to an elevated risk of neurodegenerative disease, including the link between the Epstein–Barr virus (EBV) and multiple sclerosis (Levine et al., 2023). Although not entirely new, there is growing evidence in favor of the theory that viral infections pose an environmental risk factor for neurodegenerative disorders, including additional evidence from the previously described genome-wide association study (GWAS) showing the contribution of immunological genes to AD risk (Griciuc and Tanzi, 2021). In comparison to cognitively healthy controls, a multiomic analysis found that the brains of AD patients had higher levels of genomic DNA from human herpesvirus 6A and 7 and that the quantity of these viruses was correlated with transcriptome patterns relevant to amyloid-β (Aβ) processing (Readhead et al., 2018). Parallel to this, another study found that herpes simplex virus 1 (HSV1) infection directly caused and accelerated Aβ deposition in a mouse model demonstrating Aβ pathology (Eimer et al., 2018). Collectively, these data revitalized the microbial etiology theory of AD.

Early research on histology revealed that neurofibrillary tangles in the hippocampus were infected with herpesvirus (Kittur et al., 1992). Clinical AD progression and cognitive decline are linked to serology in EBV-positive AD patients and elevated EBV IgG plasma levels (Carbone et al., 2014; Allnutt et al., 2020). Memory loss or cognitive decline in EBV positive AD patients might put dementia-prone people under stress, which can trigger immunological dysfunction and ultimately result in the replication or reactivation of EBV (Fagundes et al., 2014; Shim et al., 2017). EBV induces neurological inflammation through infected PBMCs, macrophages, and monocytes of the brain, which predicts that the virus may breach the blood–brain barrier and proliferate in the brain’s endothelial cells, resulting in a neuronal decrease in the perfusion and white matter (Kittur et al., 1992; Kanakry et al., 2016). Reduced levels of tumor necrosis factor-α (TNF-α) can lessen the tau protein and amyloid plaque hyperphosphorylation (Dezfulian, 2018). In the development of AD, tau protein hyperphosphorylation and amyloid β-protein accumulation are induced by elevated production of TNF-α in the cell line of lymphoblastoids, where EBV immortalizes B cells (Ounanian et al., 1992; Dezfulian, 2018). EBV can trigger a systemic stress immunological response through the latency and reactivation stages, which causes inflammation and, as a result, aging-related cognitive impairment (Carbone et al., 2014; Shim et al., 2017). However, there is not much information available about the pathophysiology of EBV in AD, necessitating more investigation. Several authors have proposed that the latent nature of EBV keeps the immune system persistently activated after the initial infection. This chronic inflammatory state has been shown to induce AD-like phenotypes with advancing age (Krstic et al., 2012; Carbone et al., 2014; Leonardo and Fregni, 2023). Furthermore, AD and age-related cognitive impairment have already been linked to an increase in cytokines and other early indicators of inflammation, such as alpha 1-antichymotrypsin (Carbone et al., 2014). Due to inconsistent results of the research on this subject, more investigation is necessary to determine whether AD and EBV are clearly associated. If this is proven, it would be crucial to ascertain whether EBV directly causes AD and the neurological characteristics associated with the disease. Future research in this field should be prioritized as it might aid in improving the comprehension of the function that EBV infection plays in the pathophysiology of AD.

By analyzing variations in the patterns of gene expression across the genome, DNA microarray technology can provide insights into the function of individual genes and assist in determining the most suitable targets for medication (Song et al., 2018). Finding new therapeutic targets will be made easier by integrating expression profiling approaches with microarray data. To the best of our understanding, majority of research used microarray analysis to successfully identify suitable targets for medication (Koller et al., 2017). In this work, we looked for common genetic signatures between EBV infection and AD and investigated the possible biological influence of EBV infection on the pathophysiology of AD.

**FIGURE 1:**
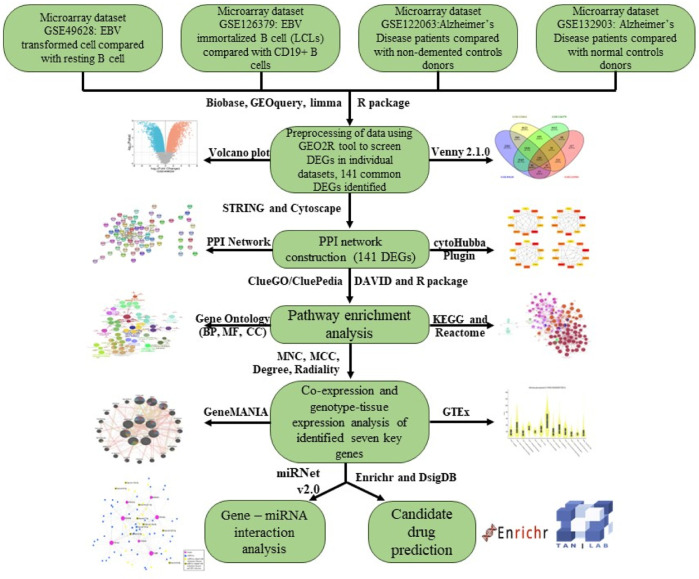
An overview of expression analysis and procedures in the current investigation utilizing bioinformatics techniques.

To begin with, we used four datasets in this study to examine the connection between EBV infection, LCLs (EBV-immortalized B cells), and AD. GSE49628 (Hansen et al., 2014), GSE126379 (Afrasiabi et al., 2019), GSE122063 (McKay et al., 2019), and GSE132903 (Piras et al., 2019) were selected from the database of Gene Expression Omnibus (GEO) for EBV, LCLs, and AD. Before we could determine whether differentially expressed genes (DEGs) were shared by all four of these datasets, we first determined which genes were differentially expressed in every dataset. Throughout the study, common DEGs were used as the primary experimental genes for gene ontology (GO), pathway analyses, and PPI network development in order to better understand genome-based biological processes. In addition, seven key genes (YWHAH, YWHAG, YWHAB, YWHAZ, MAP2K1, PPP2CA, and TUBB) were identified utilizing Cytoscape v3.9.1 for the investigation of gene regulation. Further miRNAs related to EBV and AD were identified. All things considered, our research has uncovered novel biologic processes through which EBV infection heightens AD patients’ susceptibility, as well as possible biomarkers and therapy targets (Figure 1).

## 2 Methods and materials

### 2.1 Data extraction of microarray

The GEO database offers gene expression profiles for different pathologies (Barrett et al., 2011). We used the MeSH words “Alzheimer Disease,” “EBV infected B cell,” and “human” to search for datasets pertaining to AD and EBV on the GEO (http://www.ncbi.nlm.nih.gov/geo/). The organism “*Homo sapiens*” and the study type “Expression profiling by array” were used in a subsequent filter. To ensure the quality of this research, datasets retrieved were discarded if any of the following conditions were matched.

1. Sample counts lower than 5
2. Any drug-treated patient samples

The GSE49628 (Hansen et al., 2014) dataset that sequenced using the Affymetrix Human Genome U133 Plus 2.0 Array platform contains EBV-transformed and resting B cells from three donors. The GSE126379 (Afrasiabi et al., 2019) dataset contains CD19^+^ B cells and EBV-immortalized B cells (LCLs) from five donors, which was sequenced using the Illumina HiSeq 2500 platform. The GSE132903 (Piras et al., 2019) dataset that was sequenced by using the Illumina Human HT-12 V 4.0 expression bead chip platform, contains 97 Alzheimer’s disease and 98 non-demented control donors. The GSE122063 (McKay et al., 2019) dataset contains 56 Alzheimer’s disease and 44 normal control donors, which was sequenced using the Agilent-039494 Sure Print G3 Human GE v 2 8 × 60K Microarray 039,381 platform.

### 2.2 DEG identification based on microarray data

GEO2R is an online tool that may be employed to differentiate and analyze the groups with varying expressions of gene (Barrett et al., 2013). DEGs for GSE49628, GSE126379, GSE122063, and GSE132903 were found by applying GEO2R (R v 4.2.2), which integrate the limma (v 3.54.0) package and R/Bioconductor. The *p*-value and FDR were computed using the built-in GEO2R methods including the FDR of Benjamini and Hochberg and *t*-test to discover the DEGs (Aubert et al., 2004). We set the main criteria of |log2FC| ≥ 0.5 and *p* < 0.05 to identify significant DEGs from the dataset. Upregulated DEGs were defined by log2FC ≥ 0.5, while downregulated DEGs were defined by log2FC ≤ −0.5. Venny 2.1.0 created the Venn diagram of common DEGs among these four datasets, and Bioinformatics (https://www.bioinformatics.com.cn) was applied to create the volcano maps of genes from datasets.

### 2.3 Core PPI network construction

Using STRINGv12.0 (https://string-db.org/), a search engine for the extraction of interacting genes/proteins, the DEGs were examined for protein-protein interactions (Szklarczyk et al., 2019). In order to remove inconsistent PPI interactions from the dataset, the threshold for the cutoff was established to a high confidence interaction score (≥0.4). Consequently, we were able to create a PPI network that was effective.

### 2.4 Gene ontology and pathway enrichment analysis via DAVID and ClueGO enrichment analysis

DEGs that showed STRING degree ≥ 1 were chosen for the GO and pathway analysis. We used the extremely significant web-based tool, i.e., Database for Annotation, Visualization and Integrated Discovery (DAVID) 2021 (https://david.ncifcrf.gov/), for the study of KEGG pathway and the functional annotation of GO (Huang et al., 2009; Sherman et al., 2022). It is simpler to incorporate and integrate expression data with the results of functional assessments when utilizing GO bubble plot and GO Chord to show the functional enrichment of the DEGs (Walter et al., 2015). We employed both DAVID and ClueGO tools for integrative analysis to observe the DEGs engaged in GO and pathways in detail. CluePedia v1.5.5 and ClueGO v2.5.5 were applied to the common DEGs in order to obtain all of the disease-related pathways and GO terms. ClueGO provides a fundamental ordered network of pathways or GO terms from the DEG dataset by syndicating KEGG or BioCarta pathways with GO (Bindea et al., 2009). Additionally, the analysis of pathways and molecular/biological function GO were carried out with differential genes, with *p*-value less than 0.05 being significant.

### 2.5 Intersection of PPI network and enrichment analysis of DEGs

For the purpose of conceptualizing the PPI interactions between the statistically suitable DEGs, the PPI network of STRING v12 is further transferred to Cytoscape program (v3.9.1) (Shannon et al., 2003). Using Cytoscape’s plugin CytoHubba (Version 0.1), significant PPI network nodes and hubs were investigated (Chin et al., 2014). We used four different centralities, i.e., MNC, MCC, degree, and radiality, to obtain the top five hub genes using the Cytoscape plugin CytoHubba. The set of total key genes were obtained from four centralities i.e., MNC, MCC, degree, and radiality, by using Venny 2.1.0.

### 2.6 Co-expression analysis of key genes

Based on the final key genes, GeneMANIA (http://genemania.org), an online platform for predicting gene connections and functional analysis of the identified key genes, built co-expression networks (Franz et al., 2018).

### 2.7 Gene expression analysis in genotype-tissue expression (GTEx)

The GTEx (V9) software was used to analyze the target predicted genes’ gene expression (Cheng et al., 2021). This takes us one step closer to identifying patterns of gene expression in various human brain regions.

### 2.8 Identification of miRNA interactions with key genes

miRNAs as non-coding RNAs can impair translation or damage the target mRNA. mirTarbase provided the network of miRNAs-gene (Hsu et al., 2011) *via* miRNet (Fan et al., 2016). The ID type for the organism was “Official Gene Symbol,” the tissue was “Brain,” and the host protein genes were entered into the database. After network building, all miRNAs’ relation with disease was checked through the miRNA disease database. The hypergeometric test algorithm was applied to see the miRNAs’ association with the disease.

### 2.9 Prediction of candidate drugs

In this research, assessing protein-drug interactions is crucial. Drug compounds were retrieved from DsigDB based on hub genes (Yoo et al., 2015) on Enrichr platform (Kuleshov et al., 2016). The candidate drugs were ranked from small to large using *p*-value.

## 3 Results

### 3.1 DEG identification among EBV infection, LCLs, and Alzheimer’s disease

**FIGURE 2:**
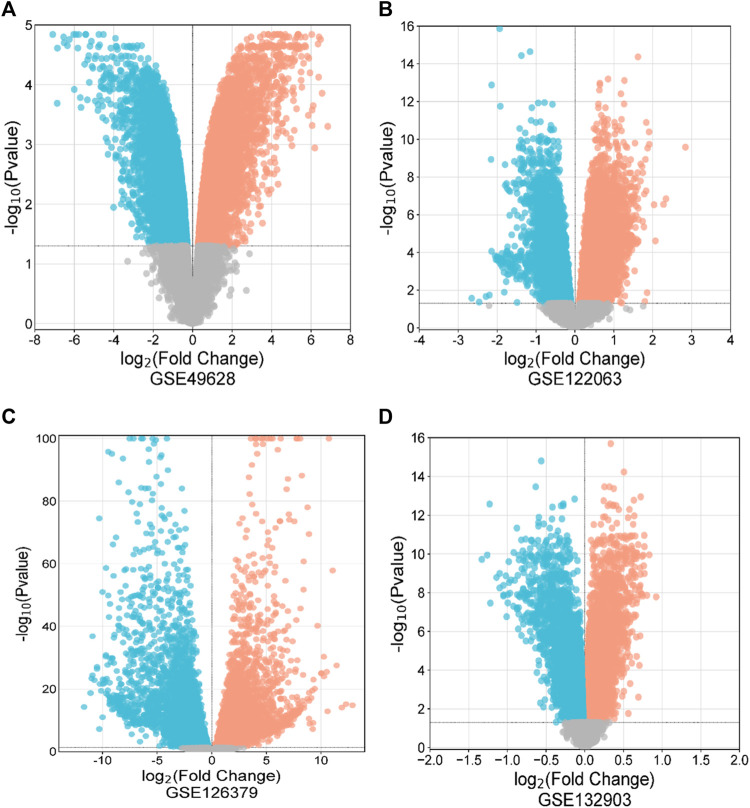
Volcano diagrams of four datasets depicting differential expression representing downregulated genes (blue), upregulated genes (red), and non-significant genes (gray). The volcano maps of GSE49628 **(A)**, GSE122063 **(B)**, GSE126379 **(C)**, and GSE132903 **(D)**.

**FIGURE 3:**
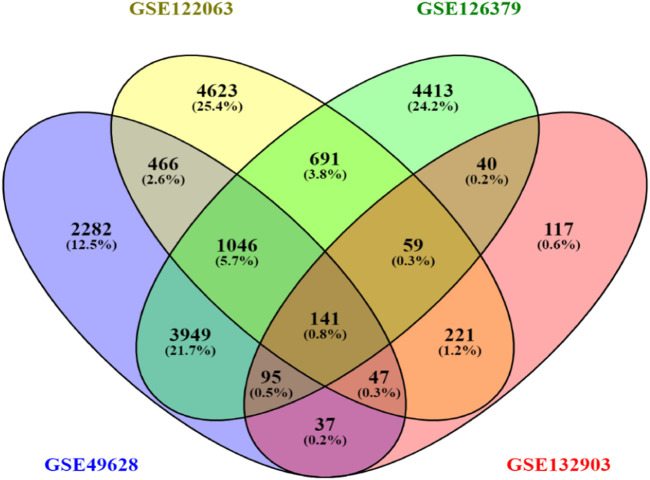
Venn diagram depicting common DEGs extracted from four datasets.

First, 8386 genes from the GSE49628 dataset showed differential expression for EBV infection, in which 3567 downregulated and 4819 upregulated genes were obtained (Figure 2A). Using the LCL dataset (GSE126379), 10,434 genes were found, which contained 5377 downregulated genes and 5057 upregulated genes (Figure 2B). We discovered 7408 and 759 DEGs for the AD datasets (GSE122063 and GSE132903), comprising 3955, 324 upregulated genes and 3453, 435 downregulated genes, respectively (Figures 2C, D). The cutoff parameters (adjusted *p*-value<0.05 and |logFC|≥ 0.5) were utilized to acquire all significant DEGs. Table 1 contains a summary of the data from various databases. Subsequently, 141 common DEGs were found and visualized using Venn diagrams (Figure 3) by taking a combination of DEGs of the EBV, LCLs, and AD datasets.

**TABLE 1:** A summary of the datasets that were used in this research, including a description of their quantitative measures and geo-features.

Name of disease	Accession of GEO	Platform of GEO	DEGs	Upregulated genes	Downregulated genes
EBV	GSE49628	GPL570	8386	4819	3567
LCLs	GSE126379	GPL16791	10,434	5057	5377
AD	GSE122063	GPL16699	7408	3955	3453
AD	GSE132903	GPL10558	759	324	435

### 3.2 PPI network analysis

**FIGURE 4:**
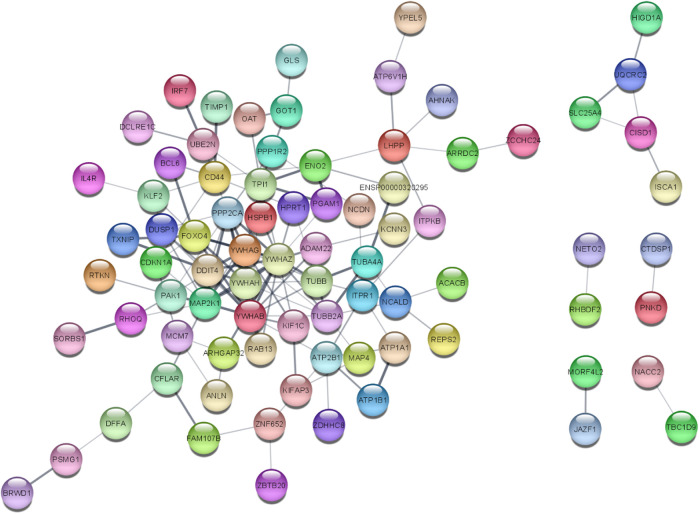
PPI network of 141 DEGs (141 nodes and 140 edges) from STRING v12.0 and visualized from Cytoscape v3.9.1. Each node represents the relevant protein or gene, and edges represent the number of associations between each node.

The STRINGv12.0 program was used to evaluate the functional and physical relationships between the proteins of differential genes. The minimal necessary interaction score was established by using a confidence value of 0.4. The significant interaction network had 141 nodes, 207 edges, and 0.379 average clustering coefficient. PPI networks are shown in Figure 4.

### 3.3 Enrichment analysis of DAVID

**FIGURE 5:**
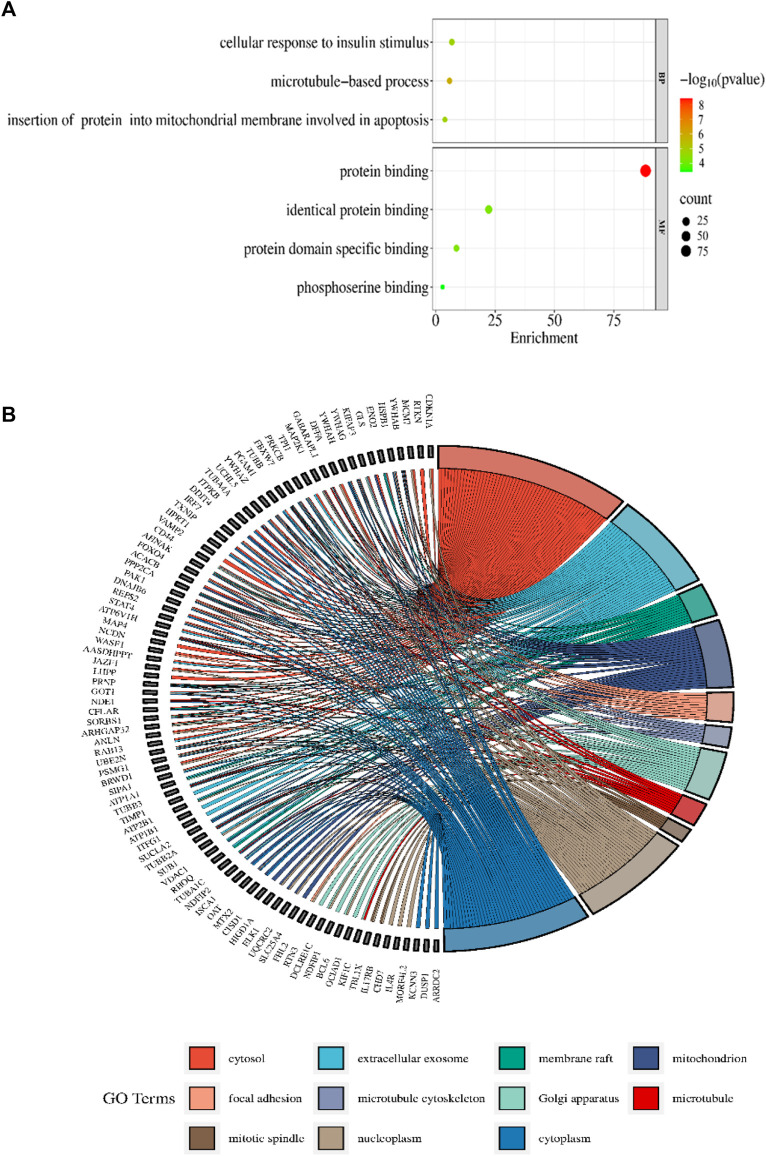
**(A)** The BP and MF of the GO terms from DAVID are illustrated in a bubble plot. The bubble size is substantially associated with the gene numbers in a specific GO term, and the -log *p*-value fluctuates with color intensity from green to red. **(B)** The GOChord figure displays the relationship among the chosen DEGs, their corresponding cellular component, and GOterms. The GO words for the various cellular components are displayed in different colors on the right side of the plot, while the DEGs involved in the various cellular components are shown on the left half. The bands of color linked a gene to a particular GO term.

The DAVID 2021 web server was utilized for the functional annotation of DEGs. Terms representing molecular function, biological processes, and signaling pathways related to KEGG were utilized to ascertain the possible GO classification and the KEGG pathway-enriched genes. Strongly enriched conditions are defined as FDR < 0.05 and EASE score (a modified Fisher exact *p*-value) ≤0.05. Through BP analysis, we discovered that differential genes from the intricate PPI network were rich in microtubule-based process (GO:0007017), apoptotic signaling pathway-related protein insert into mitochondrial membrane (GO:1900740), and cell response to insulin stimulus (GO:0032869). The MF analysis of GO demonstrated DEGs’ participation in protein domain-specific binding (GO:0019904), protein binding (GO:0005515), phosphoserine binding (GO:0050815), and identical protein binding (GO:0042802). Furthermore, we characterize the DEGs involved in the many biological pathways using the DAVID online tool based on the KEGG database (FDR <0.05; *p* < 0.05). The DEGs’ primary relationship was found via the KEGG analysis in Gap junction (hsa04540), Parkinson’s disease (hsa05012), prion disease (hsa05020), pathways of neurodegeneration (hsa05022), oocyte meiosis (hsa04114), salivary secretion (hsa04970), Alzheimer’s disease (hsa05010), cell cycle (hsa04110), and cGMP-PKG signaling pathway (hsa04022). Table 2 displays the tabulated annotated results for the specified terms. GOChord was used to plot the enrichment of the cellular components of the DEGs; GO Bubble plots were created for the MF and BP of the DEGs (Figures 5A, B).

**TABLE 2:** Terms from the GO that describe the biological processes, molecular activities, and KEGG pathways of DEGs linked to EBV and AD from DAVID.

Category	Term	Count	%	*p*-value	Fold enrichment	FDR
GOTERM_BP_DIRECT	GO:0007017∼microtubule-based process	6	5.825243	1.29E-06	31.03987241	0.001252
GOTERM_BP_DIRECT	GO:1900740∼positive regulation of protein insertion into the mitochondrial membrane involved in the apoptotic signaling pathway	4	3.883495	1.45E-05	78.63434343	0.005155
GOTERM_BP_DIRECT	GO:0032869∼cellular response to insulin stimulus	7	6.796117	1.59E-05	12.982085	0.005155
GOTERM_MF_DIRECT	GO:0005515∼protein binding	91	88.34951	3.96E-09	1.378098	1.01E-06
GOTERM_MF_DIRECT	GO:0019904∼protein domain specific binding	9	8.737864	5.14E-05	6.791696	0.005152
GOTERM_MF_DIRECT	GO:0042802∼identical protein binding	23	22.3301	6.06E-05	2.538034	0.005152
GOTERM_MF_DIRECT	GO:0050815∼phosphoserine binding	3	2.912621	3.91E-04	95.83838	0.024907
KEGG_PATHWAY	hsa04540: Gap junction	8	7.76699	8.02E-06	10.6924	0.001563
KEGG_PATHWAY	hsa05012: Parkinson disease	10	9.708738	3.54E-04	4.421671	0.016262
KEGG_PATHWAY	hsa05020: Prion disease	10	9.708738	4.17E-04	4.324134	0.016262
KEGG_PATHWAY	hsa04970: Salivary secretion	6	5.825243	0.001057	7.588157	0.029436
KEGG_PATHWAY	hsa05010: Alzheimer disease	11	10.67961	0.001277	3.369221	0.031122
KEGG_PATHWAY	hsa05022: Pathways of neurodegeneration—multiple diseases	12	11.65049	0.001893	2.96512	0.038283
KEGG_PATHWAY	hsa04110: Cell cycle	7	6.796117	0.001963	5.244045	0.038283
KEGG_PATHWAY	hsa04022: cGMP-PKG signaling pathway	7	6.796117	0.002679	4.93003	0.047487

### 3.4 Enrichment analysis of ClueGO/CluePedia

**FIGURE 6:**
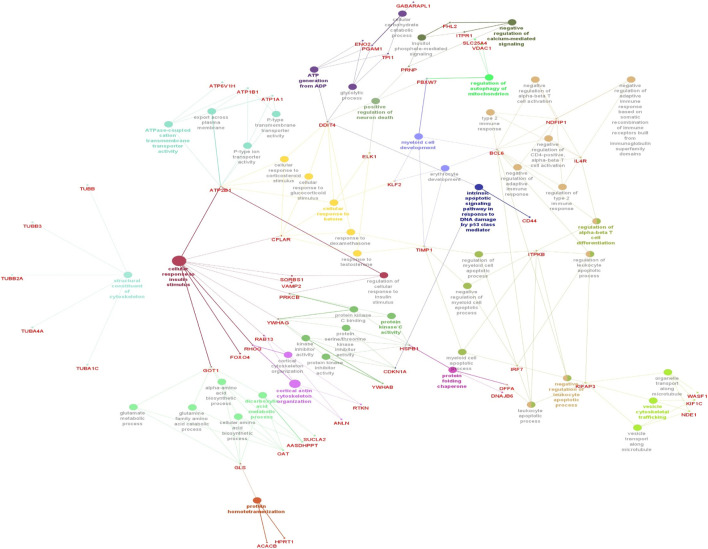
Cytoscape’s CluePedia and ClueGO plugin was applied to visualize enrichment of GO terms. The particular gene connections are employed to illustrate the biological processes (BP) and critical molecular functions (MF) associated with the DEGs. The DEG PPI network is used to deduce the findings from the enrichment analysis of MF and BP. The GO network’s connectivity is characterized by the nodes and edges with a kappa value of 0.4. The enrichment displays only GO keywords that are significant (*p*-value less than 0.05). The node size is indicated by *p* < 0.05 values. The functional class that each node is involved in is indicated by its color code. The colors stand for different biological processes and molecular functions that are engaged in the enrichment analysis. The names of each group’s MF and BP are defined by the most significant functional GO words, which are bolded. The names in red font correspond to the DEGs in each group.

**FIGURE 7:**
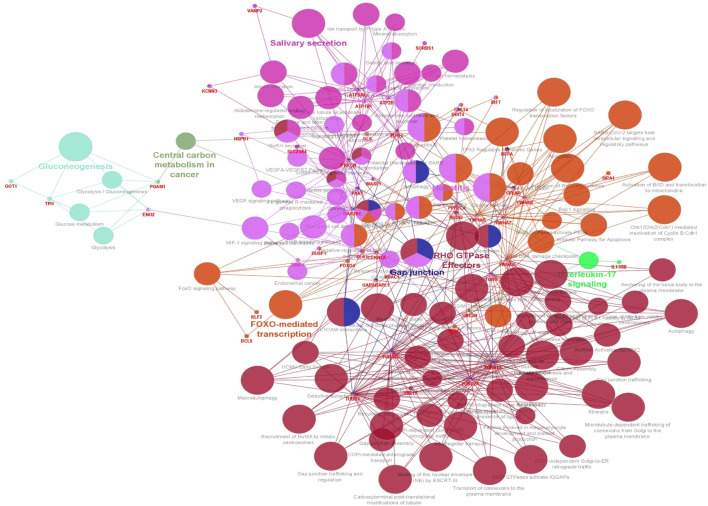
Visualization of enrichment by pathway terms is done with Cytoscape’s CluePedia and ClueGo plugin. For DEGs, the plugin offers a thorough analysis that takes into account the REACTOME and KEGG pathway. The kappa score of 0.4 indicates the pathway connectivity in the network characterized by shared edges and nodes among the DEGs. Pathways those are significant (*p*-value ≤0.05) displayed in the enrichment. The node size is indicated by values of *p* < 0.05. The particular functional class that each node is involved in is indicated by its color code. The color denotes different molecular pathways that are engaged in the DEG functional analysis that have been found. The most important functional groups of the signaling pathway are shown in bold fonts. The names in the red font correspond to the DEGs in each group.

The functionality assessment of the differential genes from the datasets was conducted by applying the ClueGO/CluePedia plugin for Cytoscape. The PPI complex network’s GO terms were made more visible with the aid of ClueGo. Figure 6 shows the terms for BP and MF in the GO analysis of the complicated PPI network. The fundamental criteria for selecting statistical parameters for ClueGO enrichment analysis were Benjamini–Hochberg correction, kappa score >0.4, and two-sided hypergeometric test with *p* < 0.05. DEGs from the intricate PPI network had BF and MF that were primarily enriched in neuronal death regulation (GO: 1901216), regulation of autophagy of mitochondrion (GO:1903146), structural constituent of cytoskeleton (GO:0005200), intrinsic apoptotic signaling pathway in DNA damage response by class mediator of p53 (GO:0042771), protein folding chaperone (GO:0044183), protein homotetramerization (GO:0051289), cortical actin cytoskeleton organization (GO:0030866), response of cell to insulin stimulus (GO:0032869), calcium-mediated signaling (GO:0050849), myeloid cell development (GO:0061515), microtubule transport (GO:0072384), ATP generation from ADP (GO:0006757), ATPase-coupled cation transmembrane transporter (GO:0019829), activity of protein kinase (GO:0004697), response of cell to ketone (GO:1901655), dicarboxylic acid metabolic process (GO:0043648), and leukocyte apoptotic process (GO:0071887) (Figure 6). ClueGO’s examination of the KEGG and REACTOME pathways revealed that some DEGs were markedly enriched in central carbon metabolism in cancer (KEGG:05230), interleukin-17 signaling (R-HAS:448424), gluconeogenesis (R-HAS:70263), Gap junction (KEGG:04540), salivary secretion (KEGG:04970), FOXO-mediated transcription (R-HAS:9614085), and RHO GTPase effectors (R-HAS:195258) (Figure 7). When considered collectively, the ClueGO enrichment data unequivocally show that DEGs alter the metabolic behavior of signaling pathways and are strongly associated with AD, accelerating the development of issues like neurodegeneration, which could eventually result in a reduction in motor nerve responsiveness. Furthermore, our bioinformatics enrichment analysis revealed several dysregulated pathways that may be crucial to the pathophysiology of EBV. To test our bioinformatics findings, however, functional validations are required.

### 3.5 Key gene identification and functional analysis

**FIGURE 8:**
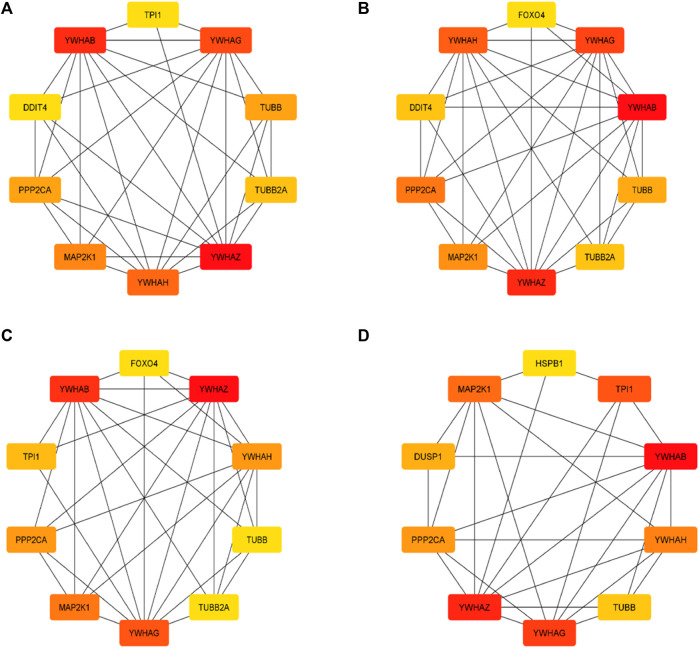
Top 10 genes from four algorithms **(A)** MNC, **(B)** MCC, **(C)** degree, **(D)** radiality of cytoHubba v0.1 from Cytoscape v3.9.1.

**FIGURE 9:**
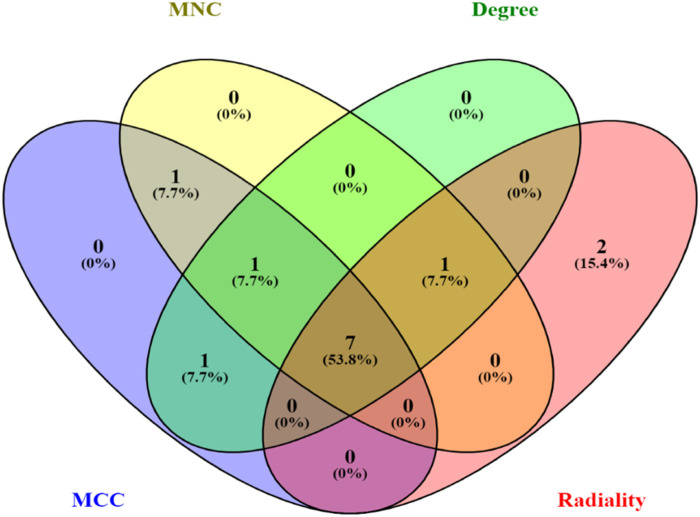
Venn diagram depicting the number of common key genes in four different algorithms of cytoHubba v0.1.

**FIGURE 10:**
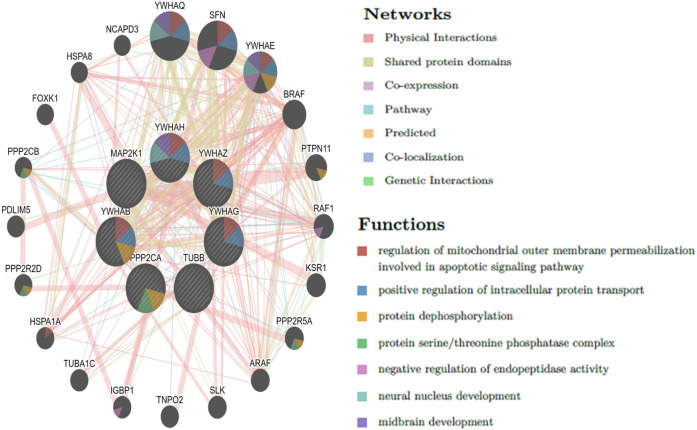
Common key genes and co-expressed genes were evaluated by applying GeneMANIA.

The top 10 genes were assessed by applying four algorithms (MNC, MCC, degree, and radiality) of the Cytoscape’s plug-in cytoHubba (Figures 8A–D). YWHAZ, YWHAG, MAP2K1, PPP2CA, YWHAH, TUBB, and YWHAB are the seven common key genes that we were able to identify by using the intersection of Venn diagrams (Figure 9). A complex gene interaction network with 51.99% physical interactions, 38.27% shared protein domains, 4.24% co-expression, 4.21% pathway, and 0.54% co-localization was built by applying GeneMANIA database to grasp the functionality of the common key genes (Figure 10). Twenty linked genes were found based on the seven major genes, indicating that they were primarily connected to positive regulation of intracellular protein transport, outer membrane permeabilization of mitochondrial involved in the apoptotic signaling pathway, protein dephosphorylation, protein serine/threonine phosphatase complex, negative regulation of endopeptidase activity, neural nucleus development, and mid-brain development. Additionally, key genes’ GO and pathway enrichment analyses revealed comparable outcomes to DEGs (Figures 5–7). In support of the above findings, it is found that 14-3-3 proteins (YWHAZ, YWHAG, YWHAH, and YWHAB) have the ability to interact with BH-3 domain-containing proteins such as BAD and BAX with both apoptotic as well as antiapoptotic properties (Zha et al., 1996; Nomura et al., 2003; Gardino and Yaffe, 2011). This dual nature of these proteins needs to be further examined in the context of EBV infection in AD patients.

### 3.6 Analysis of gene expression through genotype-tissue expression (GTEx)

**FIGURE 11:**
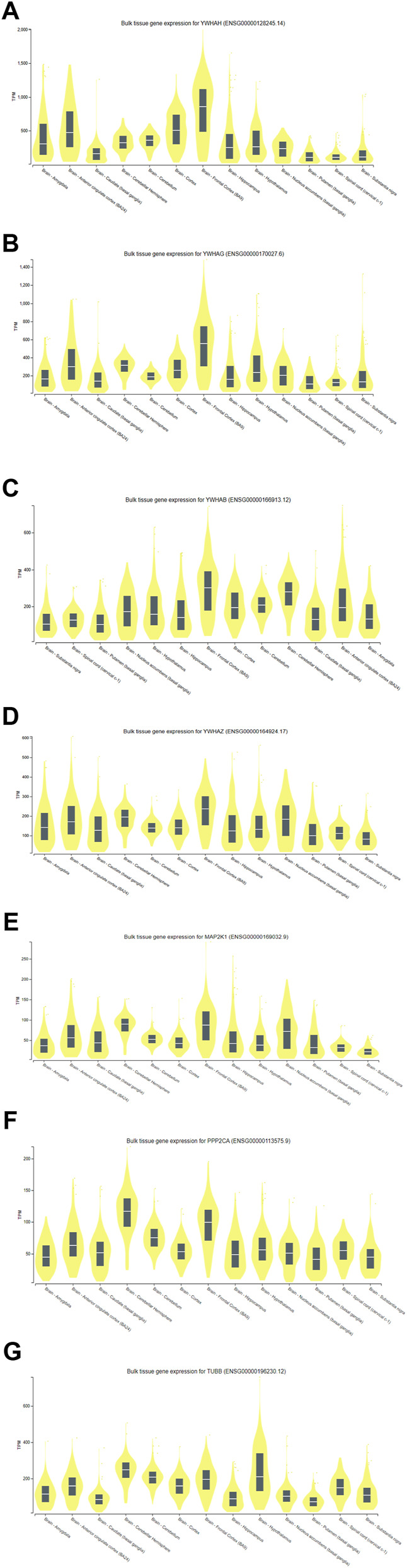
Expression analysis of **(A)** YWHAH, **(B)** YWHAG, **(C)** YWHAB, **(D)** YWHAZ, **(E)** MAP2K1, **(F)** PPP2CA, and **(G)** TUBB gene using GTEx.

With the use of the GTEx (V9) program, target-predicted gene expression was found in several regions of the human brain during the gene expression study. According to Figure 11, expression analysis of key genes (YWHAH, YWHAG, YWHAB, YWHAZ, MAP2K1, PPP2CA, and TUBB) suggested that significant expression was seen in the brain’s part, i.e., hippocampus and frontal cortex. The frontal cortex and the hippocampus were thought to be the ideal sample areas for this investigation. This demonstrated that the hippocampal and frontal cortex regions of the brain play a vital role in the functioning linked to Alzheimer’s disease. According to a study published in 2020 by Gu et al., people with AD may have impaired antiapoptotic function in their frontal cortex due to downregulation of 14-3-3γ (YWHAG) and 14-3-3η (YWHAH), which could accelerate the death of neural cells and exacerbate the pathogenesis of AD (Gu et al., 2020). The MAP2K1 gene is highly methylated in the brain of AD and could be used as a marker (Zou et al., 2023). These studies signify the role of key genes in AD.

### 3.7 Identification of miRNAs through key genes

**FIGURE 12:**
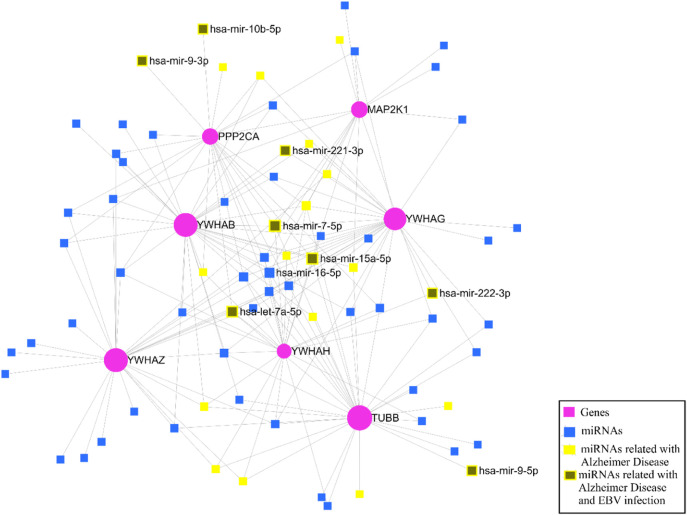
The miRNA–gene regulatory network.

After identifying the key genes, the miRNA-gene interaction was performed with the help of the miRNet v 2.0 tool which includes 81 nodes (74 miRNAs; 7 genes) and 180 edges. It has been ascertained that YWHAB, TUBB, and YWHAG have a high degree of association with the miRNAs (Figure 12). The miRNA disease association was checked, and we found that hsa-mir-15a-5p, hsa-let-7a-5p, hsa-mir-222-3p, hsa-mir-221-3p, hsa-mir-9-3p, hsa-mir-7-5p, hsa-mir-9-5p, and hsa-mir-10b-5p were associated with EBV infection and Alzheimer’s disease (Figure 12).

### 3.8 Candidate drug prediction

The top 10 potential therapeutic compounds were identified and arranged according to their *p*-value in the domains of key genes as potential pharmacological targets for EBV infection and AD using the Enrichr platform, which is built on the DSigDB database (Table 3). The four pharmaceutical compounds that were found to interact with most of the genes were glibenclamide (HL60 DOWN), 2-bromo-3-hydroxy-4-methoxybenzaldehyde (CTD 00004641), clindamycin (HL60 DOWN), and 7646-79-9 (CTD 00000928).

**TABLE 3:** List of the top 10 potential drugs for EBV and AD.

Term	*p*-value	q-value	Overlap genes
2-Bromo-3-hydroxy-4-methoxybenzaldehyde CTD 00004641	5.52E-08	2.2E-05	YWHAB, YWHAZ, YWHAG, and YWHAH
Phenethyl isothiocyanate CTD 00002443	4.31E-06	0.00085	YWHAB, YWHAZ, and YWHAH
Clindamycin HL60 DOWN	6.45E-05	0.00849	PPP2CA, YWHAB, TUBB, YWHAZ, and YWHAH
Allococaine CTD 00005697	1.01E-04	0.00999	PPP2CA, MAP2K1, and YWHAH
Diltiazem PC3 DOWN	1.98E-04	0.01566	PPP2CA, YWHAZ, and YWHAH
Paclitaxel PC3 DOWN	2.96E-04	0.01948	PPP2CA, TUBB, and YWHAZ
Glibenclamide HL60 DOWN	5.84E-04	0.03295	YWHAB, TUBB, YWHAZ, and YWHAH
Staurosporine TTD 00011086	7.47E-04	0.0369	MAP2K1 and YWHAB
Titanium dioxide CTD 00000489	1.07E-03	0.04698	TUBB and YWHAZ
7646-79-9 CTD 00000928	1.41E-03	0.04841	MAP2K1, YWHAB, TUBB, YWHAZ, and YWHAH

## 4 Discussion

Based on the many years of study, it has been found that the family of Herpesvirus is associated with neuronal degeneration. However, research on the HHV-4’s (EBV) neurotropic potential in relation to neuronal disorders like PD and AD has begun only recently. According to a number of population-based studies, those who have a chronic EBV infection and promptly reactivate it are more likely to acquire AD later in life (Huang et al., 2021). Using the constructed enrichment of GO and KEGG, we were able to ascertain the functionality of the 141 discovered DEGs in order to investigate their participation in BP, MF, and molecular pathways. These DEGs were predominantly enriched in processes involving microtubule-based process, insulin stimulus-related cellular response, and apoptotic signaling pathway-related protein regulation which insert into mitochondrial membrane. According to the MF analysis from GO, the DEGs had a substantial enrichment in protein binding, protein domain specific binding, identical protein binding, and phosphoserine binding. Additionally, the KEGG analysis disclosed that the DEGs are implicated in gap junction, Parkinson’s disease, prion disease, salivary secretion, Alzheimer’s disease, pathways of neurodegeneration—multiple diseases, cell cycle, and cGMP-PKG signaling pathway (Table 2). These results are suggesting that EBV infection might lead to neurodegeneration through mitochondrial dysfunction. Numerous investigations have yielded compelling proof that disruption of the cell cycle is a critical factor in the progression of neuronal disorders (Bonda et al., 2010; Wang et al., 2020; Yang and Gao, 2020). Moreover, it is widely known that EBV can modify the host cellular cycle that can be manipulated by a number of EBV antigens (LMPs; EBNAs, EBERs, BARTs, and EBNA-LP) (Yin et al., 2018; Awasthi et al., 2023). We used the Cytoscape’s plugin ClueGO for the interpretation of GO and KEGG pathways and created a functional ordered network of GO and pathway to further hone the molecular functions, biological processes, and pathways identified by the DAVID-GO analysis, KEGG, and STRING (Figure 5). Additionally, the plugin facilitates the visualization of the networks derived from larger, functionally organized clusters of gene (Bindea et al., 2009). ClueGO plugin was applied to identify the differentially regulated pathways and their relevant interactions of gene according to the kappa statistics and *p*-values to visualize a holistic image of the DEGs contributed in EBV infection and AD.

Among the molecular pathways and enhanced biological processes, we found that mitochondrial autophagy regulation (Tran and Reddy, 2021), neuronal death regulation (Goel et al., 2022), cytoskeleton’s structural constituent (Verma et al., 2024), intrinsic apoptotic signaling pathway in response to DNA damage by p53 class mediator (Sharma et al., 2021), protein folding chaperone (Singh et al., 2023), cellular response to insulin stimulus (De Felice et al., 2022), protein homo tetramerization, cortical actin cytoskeleton organization (Penzes and VanLeeuwen, 2011), negative regulation of calcium-mediated signaling (Tong et al., 2018), myeloid cell development (Thome et al., 2018), ATP generation from ADP (Patro et al., 2021), organelle transport along microtubule (Stevenson et al., 2016), ATPase-coupled cation transmembrane transporter (Patro et al., 2021), protein kinase activity, cellular response to ketone (Henderson, 2008), dicarboxylic acid metabolic process (Castor et al., 2020), negative regulation of leukocyte apoptotic process, leukocyte apoptotic process (Leuner et al., 2007), central carbon metabolism in cancer (Coppede, 2010), interleukin-17 signaling (Vellecco et al., 2023), gluconeogenesis (Zhang et al., 2021), Gap junction (Takeuchi et al., 2011), salivary secretion (Ashton et al., 2019), FOXO-mediated transcription (Hu et al., 2019), and RHO GTPase effectors (Bolognin et al., 2014) were found to be abnormal and are critical to neuronal degeneration in AD patients according to *p*-values and kappa statistics (Figures 6, 7). The primary EBV transforming protein, LMP-1, influences Rho GTPase signaling, which results in the development of actin stress fibers and filopodia (Puls et al., 1999). Deregulation of Rho GTPase may be a major pathogenic event that alters the characteristics of the cytoskeleton and contributes to synaptic impairments in AD (Bolognin et al., 2014). Numerous studies have reported on the role of IL-17 in the development and progression of neuro-inflammation related to AD (Kebir et al., 2007; Chen et al., 2020). Recently a research group found that EBV-infected B cells can induce the production of IL-17 from T cells (Shehab et al., 2024). Above findings suggest that differential expressed genes are significantly related to dysregulated molecular pathways and contributed to the progression of EBV-associated AD. YWHAH, YWHAG, YWHAB, YWHAZ, MAP2K1, PPP2CA, and TUBB were found to be linked with neurodegeneration risk in AD patients infected with EBV.

Of the seven key genes, four genes (YWHAH, YWHAG, YWHAB, and YWHAZ) belonged to 14-3-3 family proteins. These proteins modulate various processes in the cells, including transcription, intracellular trafficking, apoptosis, cell cycle, and autophagy. The proteins facilitate these processes by binding specific phosphothreonine- and phosphoserine-containing motifs present on various signaling proteins, including phosphatases, kinases, and transcription factors (Berg et al., 2003; Fu et al., 2003; Pozuelo-Rubio, 2012; Fan et al., 2019). These proteins found in neurofibrillary tangles for interaction with microtubule-related protein tau in AD (Hashiguchi et al., 2000; Yuan et al., 2004; Qureshi et al., 2013), despite the fact that their neurophysiological roles are still unclear (Layfield et al., 1996). PKCδ phosphorylates the tau protein, resulting in the production of binding sites for 14-3-3 protein. The Tau protein-binding site and tubulin phosphorylation is then further encouraged by the 14-3-3 protein’s binding to Tau protein *via* PKCδ. Finally, tubulin instability results from the breakdown of tau protein (Kim et al., 2010). EBV protein LMP1 regulates the PKCδ expression and might support the binding of 14-3-3 protein to the tau protein (Kung et al., 2011). Gupta and colleagues used co-IP and mass spectrometry methods to identify 14-3-3 protein (γ, ε, β, ζ, and η) as interactors of protein of EBV i.e., deneddylase (BPLF1) (Gupta et al., 2018). Numerous signaling pathways may be regulated by associations between 14-3-3 and BPLF1; potential partners in this modulation include TRIM25, ubiquitin ligases, and CUL1 (Nathan and Lal, 2020). The precise interactions that TRIM25 has with misfolded Aβ42 or p-tau before being broken down by the ubiquitin–proteasome system (UPS) are unknown. Nonetheless, a number of studies demonstrate that UPS abnormalities contribute to the pathogenesis of AD and could represent the molecular bridge connecting tau and Aβ (Cao et al., 2019). Aβ buildup, tau hyperphosphorylation, and impaired autophagy are all correlated with UPS disruption (Tai et al., 2012; Hong et al., 2014; Gadhave et al., 2016). These findings and our expression analysis in the frontal cortex and hippocampus part (Figures 11A–D) of brain suggest that 14-3-3 proteins could be the molecular target for the AD patients. A research group found that binding of 14-3-3 proteins to BAX and FOXO was impaired by direct phosphorylation of 14-3-3 by JNK (Sunayama et al., 2005). Reactive oxygen species (ROS) produced by EBV-transformed B cells allow JNK to stimulate the expression of FasL (Kim et al., 2008). This indicates that EBV modulates JNK and induces the phosphorylation of 14-3-3 proteins to release BAX and BAD protein for apoptosis, which leads to further neurodegeneration. Further investigation is needed in the context of whether EBV modulates 14-3-3 proteins directly or indirectly.

The MAP2K1 gene codes for the MAP2K1 protein, a signaling protein of mitogen-activated protein kinases (MAPKs) that controlled nearly every cellular activity that was stimulated, such as stress responses, proliferation, and differentiation (Keshet and Seger, 2010; Flores et al., 2019). Numerous pathological processes, including neuronal disorders, i.e., HD, AD, MS, ischemia, and cerebral hypoxia, were influenced by the dysregulation of these kinases (Collins et al., 2015; Zou et al., 2023). MAPK/ERK hyperactivity is brought on by viral infection, specifically EBV infection (Roberts and Cooper, 1998), that could be mediated by DUSP-8 and DUSP-6 (MKP-3) downregulation. EBV antigens, such as LMP-1 (Roberts and Cooper, 1998) and EBNA2 (Rosato et al., 2012), are the most obvious source of the virus’s ability to upregulate MAPK/ERK. Several studies have demonstrated a connection between the activation of MAPKs and the build-up of hyperphosphorylated tau protein and pathogenic beta-amyloid (Aβ) aggregates in neuroinflammation (Ahmed et al., 2020) and neurofibrillary plaques (Kirouac et al., 2017; Sun and Nan, 2017).

Tubulin Beta Class, or TUBB, was also found to be differently expressed and connected to pathways, leading to neurofibrillary tangles in AD (Liang et al., 2008). TUBB could be a peripheral biomarker for AD and could be helpful in developing treatment strategies to treat AD (Rahman et al., 2020). There are no articles discussing TUBB’s function in EBV association in AD, which emphasizes the need for more research on this topic.

The PPP2CA (protein phosphatase 2, catalytic subunit, alpha) gene encodes an enzyme called protein phosphatase 2 (PP2A) (Jones et al., 1993; Virshup and Shenolikar, 2009). The EBV encodes EBNA-LP, which binds to PP2A to prevent its downstream proapoptotic signaling and causes B-cell immortalization and transformation (Garibal et al., 2007). In the brain, PP2A dephosphorylates tau, which has a preventative effect. The dynamics of microtubules can alter PP2A’s affinity for tau protein. Numerous anomalies leading to the inability of this enzyme to operate have been documented in various research studies on AD. These include lower amounts of PP2A regulatory or catalytic subunits, changed post-translational modification targets, phosphorylation of PP2A, or decreased PP2A enzymatic activity (Mandelkow et al., 1995; Bloom, 2014). According to Liu et al.’s findings from 2004, PP2A, which controls tau protein phosphorylation at several locations, is the primary tau phosphatase among all phosphatases. The results of these investigations propose that PP2A might be a critical target for AD (Liu et al., 2004). According to this *in silico* expression study of the major genes, brain’s frontal cortex and hippocampus exhibit considerable expression (Figure 11). The frontal cortex and hippocampus were selected as the study’s target sample areas. This demonstrated that the brain’s frontal cortex and hippocampus have essential functional processes associated with Alzheimer’s disease.

To find the hub genes’ post-transcriptional regulators, we also conducted the miRNAs-gene association analysis. As more than 30 microRNAs have been found to target YWHAB, it has been determined that it plays a substantial function in the network. Furthermore, it was found that microRNAs like hsa-mir-15a-5p, hsa-let-7a-5p, hsa-mir-221-3p, hsa-mir-222-3p, hsa-mir-7-5p, hsa-mir-9-5p, hsa-mir-9-3p, and hsa-mir-10b-5p were associated with EBV infection and Alzheimer’s disease (Figure 12). This revealed that the regulation of the most key genes is primarily controlled by three miRNAs—hsa-mir-15a-5p, hsa-let-7a-5p, and hsa-mir-7-5p—which are also linked to EBV infection. Ye et al., 2020 performed gene co-expression network analysis and found hsa-mir-15a-5p as the marker for aging and cognitive disorder in elders (Ye et al., 2020). A research group constructed an artificial neural network and found that has-mir-let-7a-5p could be a diagnostic biomarker for Alzheimer’s disease (Talemi et al., 2023). Another study group observed elevated hsa-miR-7-5p expression connected to the NLRP3 inflammasome’s activity, which is a key molecule in the neuroinflammatory pathway of AD (La Rosa et al., 2021). Other than AD, these miRNAs are important in neuronal degenerative disorders such as PD and multiple sclerosis (MS) (Hecker et al., 2013; Yang Y. et al., 2021; Sun et al., 2022).

The DSigDB database was utilized to predict possible medications using seven hub genes. In the predicted top 10 drugs, some of them were found directly to be able to cure neurodegenerative disorders. It was recently shown that treating toxoplasmosis with antibiotics like clindamycin was linked to a lower incidence of dementia (Yang HY. et al., 2021). Another study shows that the calcium channel blocker diltiazem may have a protective effect against Huntington’s disease caused by quinolinic acid by inhibiting various neuroinflammatory pathways (Kalonia et al., 2011). Some research studies have shown that antimitotic MT-stabilizing agents, such as paclitaxel, may be used as medications to stop or delay the development of tauopathies and prevent AD (Shemesh and Spira, 2011; Cross et al., 2021). With type 2 diabetes and sporadic Alzheimer’s-like illness in rat experiments, glibenclamide (GBC) was found to reduce hippocampus neuroinflammation and cognitive impairment. To determine whether GBC therapy is linked to cognitive improvement in patients with sporadic AD, more human research is required (Esmaeili et al., 2020).

Therefore, it was determined that 14-3-3 proteins (YWHAH, YWHAG, YWHAB, YWHAZ), MAP2K1, PPP2CA, and TUBB) were among the most significant results as they are largely implicated in all significant pathways linked to EBV infection and AD. In light of the fact that this is a novel study finding that highlights its considerable expression in AD patients, it may therefore result in a better biomarker.

Certainly, there is a limitation in every *in silico* study. On one side, there was a scarcity of information regarding Alzheimer’s disease with EBV infection to further corroborate our findings. On the other side, as our results were derived only from bioinformatics analysis, additional *in vitro* and *in vivo* research is needed to confirm the roles of important genes and possible medications. Our predicted drugs could be the target of the products of key genes linked with late-onset AD due to EBV infection but the mechanisms underlying disease genetics and drug actions require further elucidation before we can propose effective drug targets to combat late-onset AD. More collaborative efforts are essential to overcome these hurdles and fully leverage the potential of bioinformatics in healthcare.

## 5 Conclusion

In conclusion, we obtained seven key genes (YWHAH, YWHAG, YWHAB, YWHAZ, MAP2K1, PPP2CA, and TUBB), most associated three miRNAs (hsa-mir-15a-5p, hsa-let-7a-5p, and hsa-mir-7-5p) to key genes, and top 10 potential therapeutic targets for AD patients by utilizing a comprehensive bioinformatics approach. This study explores possible molecular processes that underlie the impact of EBV infection in Alzheimer’s disease patients. These results might provide a new perspective on treating Alzheimer’s disease. Clinically, these insights suggest that monitoring EBV infection markers and related molecular changes could become part of the diagnostic process for AD. These could be utilized for early screening of high-risk populations and for initiating disease management and drug intervention during the early stages. This strategy might include developing drugs or gene therapies aimed at the identified miRNAs and their target genes, potentially slowing or altering the course of AD in patients with EBV infection. We expect to carry out clinical investigations and molecular studies to validate the findings of this study.

## Data Availability

The raw data supporting the conclusions of this article will be made available by the authors, without undue reservation.
